# A case report of pulmonary tumor thrombotic microangiopathy (PTTM) caused by esophageal squamous cell carcinoma

**DOI:** 10.1007/s10388-013-0382-8

**Published:** 2013-07-13

**Authors:** Takeshi Fujishiro, Kiyohiko Shuto, Toru Shiratori, Tuguaki Kono, Yasunori Akutsu, Masaya Uesato, Isamu Hoshino, Kentaro Murakami, Shunsuke Imanishi, Toru Tochigi, Yoko Yonemori, Hisahiro Matsubara

**Affiliations:** 1 Department of Frontier Surgery, Graduate School of Medicine, Chiba University, Inohana 1-8-1, Chuo-ward, Chiba, Chiba 260-8677 Japan; 2 Department of Surgery, Teikyo University Chiba Medical Center, Ichihara, Japan; 3 Department of Diagnostic Pathology, Graduate School of Medicine, Chiba University, Chiba, Japan

**Keywords:** Pulmonary tumor thrombotic microangiopathy, PTTM, Esophageal squamous cell carcinoma

## Abstract

A 67-year-old male was referred to our hospital after being diagnosed with esophageal squamous cell carcinoma of the middle thoracic esophagus. The clinical stage was T1b(sm)N4M1 cStage IVb, so he was admitted to our hospital for systemic chemotherapy. He had sustained fever and a dry cough. Chest computed tomography showed the presence of irregular shadows, and unidentified respiratory insufficiency had progressed. A transbronchial lung biopsy revealed a pulmonary artery tumor embolus of esophageal squamous cell carcinoma. He developed DIC and died of respiratory failure on the 19th hospital day. The postmortem autopsy detected pulmonary tumor thrombotic microangiopathy accompanied by esophageal squamous cell carcinoma.

## Introduction

Pulmonary tumor thrombotic microangiopathy (PTTM), defined by von Harvey et al. [1] in 1990, has been known as a rare but fatal pulmonary complication related to cancer. It is histopathologically characterized by widespread tumor cell emboli associated with fibrocellular intimal proliferation and pulmonary arteriole stenosis or obstruction. To date, PTTM associated with esophageal squamous cell carcinoma is considered to be very rare. We herein present a case report of PTTM as a complication of esophageal squamous cell carcinoma.

## Case report

**Fig. 1:**
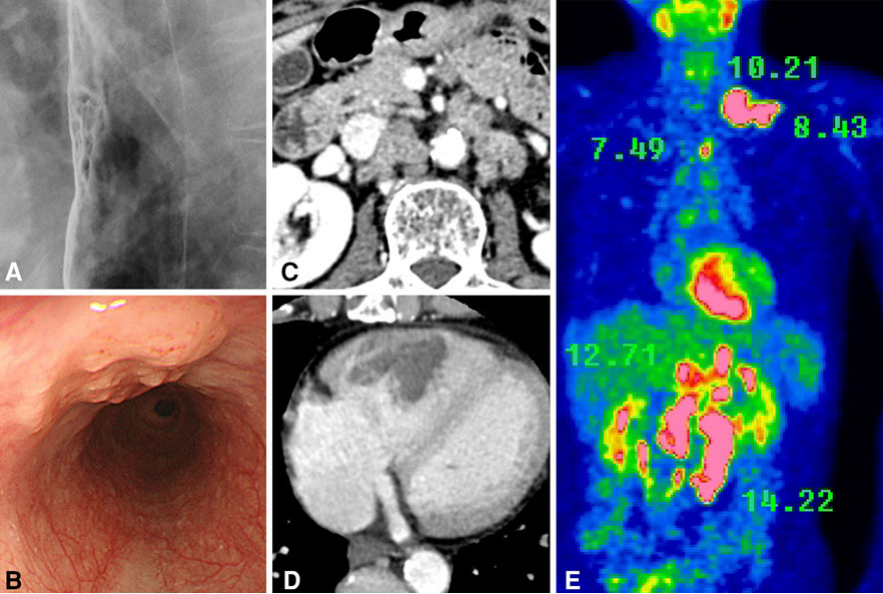
Esophageal squamous cell carcinoma was detected in the middle thoracic esophagus (**a**, **b**). Contrast-enhanced CT showed the multiple para-aortic lymph node metastases and cardiac right ventricle metastasis (**c** **d**). FDG-PET showed multiple high accumulations of FDG (shows SUV counts) (**e**)

A 67-year-old healthy male was referred to our hospital after being diagnosed with esophageal cancer. Upper gastrointestinal endoscopy revealed poorly differentiated squamous cell carcinoma of the middle thoracic esophagus. Contrast-enhanced computed tomography (CT) and ^18^F-2-fluoro-2-deoxy-d-glucose-positron emission tomography (FDG-PET) showed multiple supraclavical, mediastinal and para-aortic lymph node metastases, and cardiac right ventricular metastasis (Fig. 1). The clinical stage was T1b(sm)N4M1, cStage IVb according to the Japanese Classification of Esophageal Cancer [2]. The patient was admitted to our hospital for systemic chemotherapy in March 2010.

**Fig. 2:**
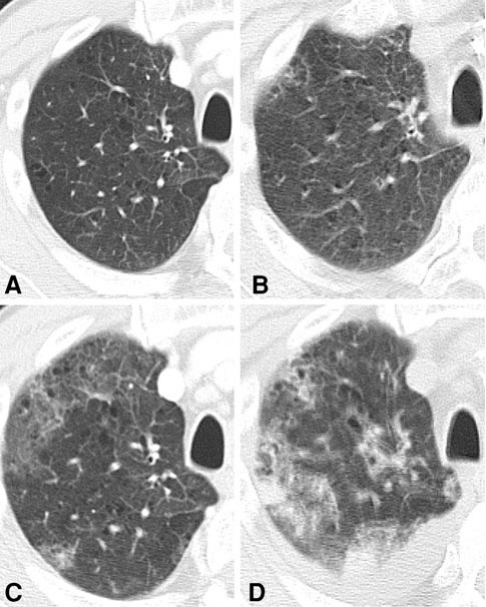
The progressive multiple lung shadows were detected with chest CT. Twelve days before admission (**a**), on the 2nd hospital day (**b**), 11th day (**c**) and 19th day (**d**)

At the time of admission, he had sustained fever of 38 °C and a dry cough. Chest CT on the 2nd hospital day showed the presence of irregular shadows under the bilateral pleura, then the shadows gradually increased and respiratory failure of unknown origin progressed rapidly (Fig. 2).

Transthoracic echocardiography on the 9th hospital day showed, in addition to metastasis in the right ventricle, slight pulmonary hypertension. A transbronchial lung biopsy revealed pulmonary arterial tumor emboli of squamous cell carcinoma. The disease progressed to disseminated intravascular coagulation (DIC), and we added treatment for DIC, but the patient died of respiratory failure on the 19th hospital day.

## Pathological findings

**Fig. 3:**
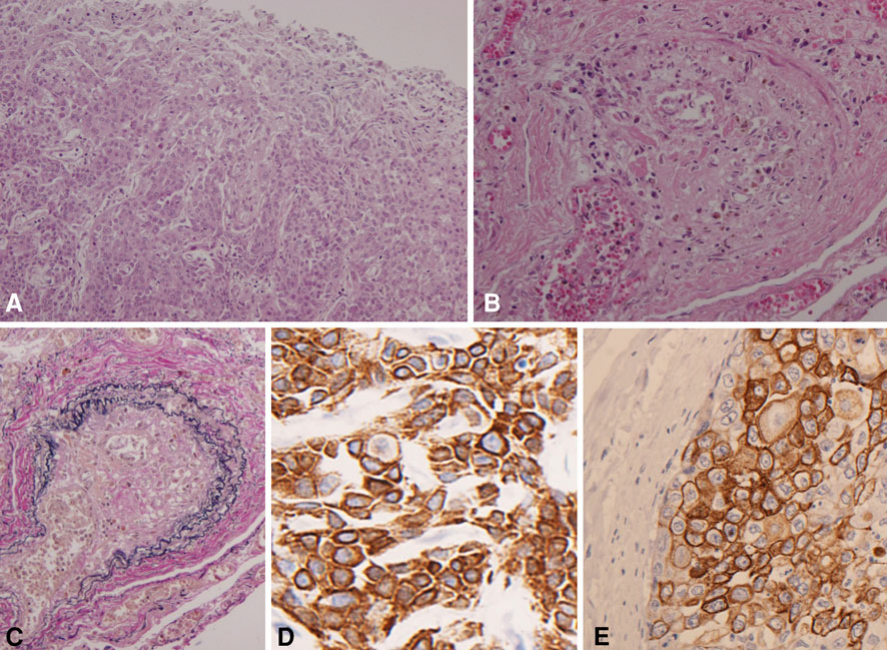
Microscopic examination. The primary tumor of the esophagus (**a** H&E ×100). Multiple tumor emboli and clot formation were shown in the pulmonary arterioles (**b** H&E ×100). EVG staining of the pulmonary arterioles showed concentric fibrocellular intimal proliferation with narrowing of the lumen (**c** EVG ×100). Immunostaining for CK5/6 proved that both the positivity rate and staining intensity were high (**d** primary tumor of the esophagus, CK5/6 ×400, **e** tumor cells in the pulmonary arteriole, CK5/6 ×400)

With the approval of the patient's family, a postmortem autopsy was performed. Macroscopically, the esophageal primary lesion was type 0–IIc with a 2.2-cm longer axis in the middle thoracic esophagus; clinicopathologically, it was poorly differentiated squamous cell carcinoma, sm3, ly3, v0. In the bilateral lungs, macroscopically mosaic-shaped bleedings, small infarctions, edema and non-segmental white fibrosis were diffusely spread. No macroscopic thrombi were detected in the pulmonary arteries or their main branches. A microscopic examination of the lungs revealed multiple tumor emboli and clot formation in the pulmonary arterioles. Elastica van Gieson (EVG) staining showed fibrocellular intimal proliferation. These findings were consistent with the typical view of PTTM. We added the immunostaining of cytokeratin (CK) 5/6, proving that tumor cells in both the primary tumor and pulmonary arteriole had a high positivity rate and staining intensity (Fig. 3). No apparent distant metastases or lymphangitis carcinomatosa was detected in the lungs. However, massive lymph node metastases were detected in the mediastinum, para-aorta and left supraclavicle, and distant metastases were detected in the pancreas head and cardiac right ventricle wall.

**Fig. 4:**
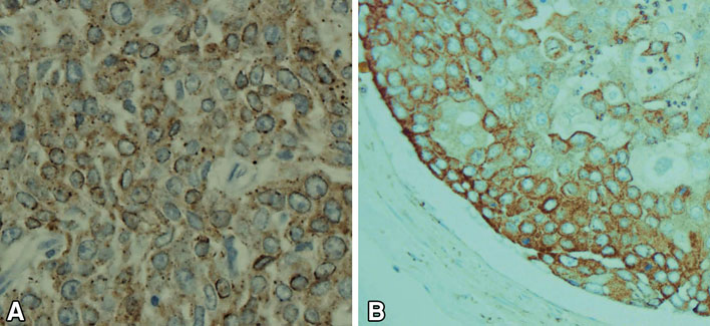
Immunostaining for VEGF. VEGF-positive tumor cells showed diffuse cytoplasmic staining (**a** primary tumor of the esophagus, VEGF ×400; **b** tumor cells in the pulmonary arteriole, VEGF ×400)

Additional immunostaining for vascular endothelial growth factor (rabbit anti-VEGF, RB-9031-R7, Thermo Scientific, USA) showed that VEGF-positive cancer cells occupied the majority of the sites of both the primary tumor and the tumor emboli in the pulmonary arterioles (Fig. 4).

In addition, there was an early gastric cancer of 7 mm diameter on the lesser curvature of the lower body, which was a well-differentiated tubular adenocarcinoma with no lymph node metastasis. The final diagnosis from the autopsy was PTTM caused by squamous cell carcinoma of the esophagus.

## Discussion

PTTM is a rare but fatal pulmonary complication related to cancer that was first described by von Harvey et al. [1]. According to his report, PTTM is a subtype of pulmonary tumor embolism (PTE) and observed in 0.9–3.3 % of autopsies of patients with malignant tumors. Until this description of PTTM in 1990, the definition of so-called pulmonary thromboembolism (PTE) had been unclear. PTTM is characterized by fibrocellular intimal proliferation and focal hypercoagulability, which are secondary to the tumor cell emboli of the pulmonary small arterioles. It results in pulmonary arteriole stenosis or obstruction, and then the patient shows severe dyspnea and pulmonary hypertension. More than half of the cases (55.6 %) are eventually complicated with DIC [3]. The prognosis of PTTM is very poor, and typically patients die within a few days after admission [4–7] without receiving the diagnosis of PTTM while alive.

We performed a literature search on PTTM using MEDLINE and the Japan Medical Abstracts Society (JAMAS) from 1990 to March 2013. Including our case, 103 cases (51 overseas and 52 domestic cases) have been reported since Harvey published the definition in 1990. Of note, there is no gender-related difference in the incidence (53 males and 50 females), and the mean age of patients is 55 years (range 11–87 years old).

According to our research of the past literature, the mean length from onset to admission is about 1 month, and the disease progresses rapidly. The median survival of patients who died was only 5 days from admission.

The main clinical problem is the difficulty of diagnosising PTTM. Most of the patients die of respiratory failure without a diagnosis of PTTM. Only 7 of 103 reported patients were pathologically diagnosed with PTTM while alive. In the previous literature, the pathological diagnosis of PTTM was made by transbronchial lung biopsy (TBLB) [5, 8], pulmonary segmental resection [9, 10] and CT-guided pulmonary biopsy [11]. On the other hand, in our case, the pathological diagnosis by TBLB was PTE associated with squamous cell carcinoma; it was not possible to diagnose the PTTM while the patient was alive.

The most frequent primary cancer complicated by PTTM is gastric cancer (58 of the 103 cases), followed by lung cancer (10 cases), breast cancer (7 cases), cancer of unknown primary (5 cases), ovarian cancer (5 cases) and bladder cancer (4 cases). Including our case, there have been only two reported cases associated with esophageal squamous cell carcinoma.

With regard to the categorization of the tissue type, adenocarcinoma was the most common, in particular poorly differentiated adenocarcinoma (including 21 cases of signet cell carcinoma and 7 cases of mucinous types) accounted for 82.8 % of the gastric cancers complicated by PTTM. Four cases with early gastric cancer in the mucosa or submucosa were reported in the previous PTTM literature [12–15], whereas there have only been five reported cases of squamous cell carcinoma (two cases each of esophageal cancer [16] and lung cancer, and one case of uterine cervical cancer).

To our knowledge, a definitive treatment for PTTM has not been established, and no studies reported performing surgery for PTTM. Thirteen of the 103 reported patients received systemic chemotherapy [5, 8, 9, 11, 16, 17], and 5 of these patients survived longer than 3 months [5, 8, 10, 11]. Comparing the survivors and non-survivors, ten patients who had dyspnea related to PTTM before treatment had a poor prognosis (acute mortality of 80 %).

Immunohistochemical examination of cancer cells in the pulmonary arterioles was described in some reports. The expression of vascular endothelial growth factor (VEGF) was enhanced [6, 18–21]; platelet-derived growth factor (PDGF) [18] and tissue factor (TF) [6, 14, 19, 22] also showed the same trend. According to some of the recently reported studies, plasma VEGF was significantly higher [16, 21]. It has been demonstrated that VEGF is a major regulator of angiogenesis. In addition, VEGF induces an increase in the blood vessel endothelium [23, 24]. TF not only causes an increase of the promotion of the vessel endothelium, but also activates clot formation [25, 26]. Furthermore, VEGF acts on the endothelial cells and leads to upregulation of TF mRNA and protein expression on the cell surface, so that the overexpression of VEGF indirectly leads to clot formation by promoting TF expression [27]. PDGF is related to the migration of fibroblasts and vascular smooth muscle cells, and an increase in the proliferation of vascular smooth muscle cells [28]. It has been suggested that these cytokines may participate in the clinicopathological conditions associated with PTTM, and the administration of molecular-targeted drugs such as bevacizumab (an anti-VEGF receptor antibody) might be effective for non-symptomatic PTTM.

In conclusion, PTTM tends to be a common complication of gastric cancer, especially poorly differentiated adenocarcinoma, and it is often accompanied by massive lymphatic invasion and systemic lymph node metastases. Clinically, PTTM is rapidly progressive and has a poor prognosis. The use of chemotherapy may extend the survival of patients. However, it does not appear to be useful in patients with dyspnea prior to treatment.
